# New modification of modified bentall procedure (A single centre experience)

**DOI:** 10.12669/pjms.316.7855

**Published:** 2015

**Authors:** Ghulam Hussain, Naseem Ahmad, Sohail Ahmad, Mirza Ahmad Raza Baig, Sara Zaheer, Aamir Furkan

**Affiliations:** 1 Ghulam Hussain, FCPS Cardiac Surgery. Senior Registrar Cardiac Surgery, Cardiac Surgery Department, Ch. Pervaiz Elahi Institute of Cardiology (CPEIC), Multan, Pakistan; 2 Naseem Ahmad, FCPS Cardiac Surgery. Assistant Professor of Cardiac Surgery, Cardiac Surgery Department, Ch. Pervaiz Elahi Institute of Cardiology (CPEIC), Multan, Pakistan; 3 Sohail Ahmad, M.Sc Pain Medicine. Assistant Professor of Anesthesia, Cardiac Surgery Department, Ch. Pervaiz Elahi Institute of Cardiology (CPEIC), Multan, Pakistan; 4 Mirza Ahmad Raza Baig, B.Sc Hons. Cardiac Perfusion Technology, Clinical Perfusionist, Cardiac Surgery Department, Ch. Pervaiz Elahi Institute of Cardiology (CPEIC), Multan, Pakistan; 5 Sara Zaheer, MBBS. Woman Medical Officer, Civil Hospital Multan, Pakistan; 6 Aamir Furkan, FCPS Anesthesia. Medical Officer, Cardiac Surgery Department, Ch. Pervaiz Elahi Institute of Cardiology (CPEIC), Multan, Pakistan

**Keywords:** Bleeding, Modified Bentall Procedure

## Abstract

**Background and Objectives:**

Modified Bentall procedure has become a gold standard in the treatment of combined aortic root and aortic valve diseases. Bleeding is an important predictor of morbidity and mortality after the Bentall operation. Our objective was to evaluate the early outcomes of Modified Button-Bentall procedure with cuff technique for aortic root replacement surgery regarding hemostasis.

**Methods:**

A total number of 32 patients who underwent elective Bentall operation from January 2008 to December 2014 were included in the study. In 18 patients (Group I) modified Button-Bentall procedure with formation of cuff was used and in 14 patients (Group II) Modified Button technique without cuff formation was used for aortic root replacement. Data was analyzed using SPSS V16. Chi-square test, Fisher’s Exact test and independent sample t-test was used to analyze Qualitative and Quantitative variables.

**Results:**

Three patients in Group II and two patients in group I was in congestive cardiac failure pre-operatively. Out of thirty two patients two patients were having Aortic root dissection one in each group. Total bypass time and cross-clamp time were significantly high in Group I. There was no significant difference regarding duration of inotropic support, ventilation time, ICU stay and hospital stay time in patients of Group I and Group II. But post-op Chest drainage was very high in Group II 1158+451.25 ml versus 488.89+168.27 ml in group I (p-value <0.0001). There was one in hospital death in Group II.

**Conclusions:**

Formation of cuff of remnant of aorta during proximal anastomosis results in significant reduction in post-operative bleeding and was better in hospital outcomes.

## INTRODUCTION

Since the original description of Bentall procedure in 1968 by Bentall and De Bono^1^ and Modified Button Technique in 1981,^2^ aortic root replacement technique with a composite valve Dacron graft has become a gold standard in the treatment of combined aortic root and aortic valve diseases. Modified Bentall technique have many beneficial effects over the original Bentall Technique like reduced tension on button coronary anastomosis, prevention of excessive bleeding and development of false aneurysms, prevention of kinking of coronary arteries, decreased operative time, and performance of complete aortic root replacement with less morbidity and mortality rate.^3^-^5^

The new generation of Dacron grafts and the various technical modifications in Bentall procedure that were aimed to reduce tension on coronary anastomoses buttons have effectively prevented bleeding from the anastomoses between the graft and coronary anastomosis.^6^,^7^ Despite all these, early mortality and non-cardiac morbidity rates are still higher than for other cardiac surgical procedures, and these rates correlate closely with excessive bleeding during operation.^8^-^10^ Bleeding is still a devastating complication after root replacement surgery.^7^

In this observational study, we report our experience in a 5-year series of electively performed modified button-Bentall operations, in which we made cuff of remnant of aorta over the prosthetic graft during aortic root replacement. Our main focus was to reduce perioperative bleeding and improvement of in-hospital outcomes and to evaluate the early outcomes of Modified Button Bentall procedure with cuff technique for aortic root replacement surgery regarding hemostasis.

## METHODS

It was a retrospective randomized observational study conducted in Cardiac Surgery department of Ch. Pervaiz Elahi Institute of Cardiology, Multan, Pakistan. The data of patients who underwent composite graft replacement of the aortic valve and ascending aorta with reimplantation of the coronary arteries either with cuff formation or without cuff formation from January 2008 through December 2014 were retrieved retrospectively from cardiac surgery data base of the hospital. There were a total number of 32 patients, in 18 patients (Group I) modified Button-Bentall procedure with formation of cuff was used and in 14 patients (Group II) modified Button technique without cuff formation was used for aortic root replacement.

### Surgical Technique

All operations were carried out through median sternotomy and standard cardiopulmonary bypass using two stage single venous right atrial cannula and a straight tip Aortic was established in every patient. All operations were carried out using moderate to severe hypothermia (temperature range 28-26°C) Cold blood cardioplegia was used for myocardial protection, topical cooling of heart was maintained with iced slush throughout the procedure.

After going on bypass, the primary tear, or Aneurysm transected 2 cm below the aortic cross-clamp, the ascending aorta was fully transected at that level and prepared for distal anastomosis. The aortic valve was excised and the annulus size was measured. The proximal end of the composite valved graft was attached to the aortic wall tissue (cuff) or LVOT. In the Cuff Technique we used 8 to 9 mm Native Aortic cuff to reinforce our proximal anastomosis, while in non-cuff technique composite graft was directly anastomosed to LVOT. Coronary buttons were fashion with at least 5mm aortic wall tissue around ostia. Left coronary buttons were anastomosed with opening made in composite grafts with 5/0 prolene stitch. Then, the conduit was pressurized with cardioplegia, and the right ventricle was dilated (short clamping of the venous line) to determine the exact position of the right coronary ostium to the graft. At that time any significant leaking at the level of the left coronary ostium anastomosis was identified. The right coronary ostium anastomosis to the graft was performed in the same fashion. After that graft length was measured and the distal anastomosis of the conduit to distal aorta was performed with a continuous 4-0 polypropylene suture. Excellent haemostasis was obtained and the patient was weaned from cardiopulmonary bypass.

### Statistical Analysis

Data was analyzed using SPSS. V16. The preoperative, operative and postoperative characteristics were summarized using means and standard deviation for the numeric variables. The groups were compared using Student’s t-test for numeric variables. Chi-square test and Fishers Exact test were used to analyze categorical variables

## RESULTS

Pre-operative characteristics of patients are shown in Table-I. There was no significant difference regarding demographic and Echocardiographic characteristics of patients. Three patients in Group II and two patients in group I were in congestive cardiac failure pre-operatively (Table-I). Out of thirty two patients two patients were of having Aortic root dissection one in each group.

**Table-I:** Comparison of Pre-operative Characteristics.

Variable		Cuff Group (Group I)	Without Cuff (Group II)	p-value
Age (years) (mean±S.D)		60.50±10.49	61.36 ± 11.47	0.83
Gender (male %)		12 (66.7)	10 (71.4)	1.00
NYHA* Class II, III (%)		5 (27.8), 13 (72.2)	6 (42.9), 8 (57.1)	0.465
Pre-op Cardiac Failure (%)		2 (11.1)	3 (21.4)	0.63
Ejection Fraction (mean±S.D)		55.55 ± 9.21	53.57 ±13.27	0.63
Type of Disease	Aneurysm	17 (94.4%)	13 (7.1%)	0.85
	dissection	01 (05.6%)	01 (92.9%)	

* NYHA= New York Heart Association.

Total bypass time and cross-clamp time were significantly high in Group I. there was no significant difference regarding duration of inotropic support, ventilation time, ICU stay and hospital stay time in patients of Group I and Group II (Table-II). However the post-op Chest drainage was very high in Group II, the maximum drainage was 2300 ml and that patient was off Group II and the patient expired ultimately because of hemodynamic instability. Rate of re-opening and immediate in hospital operative mortality was also high in Group II patients (see Table-II). So according to the results of this study, Aortic root replacement using cuff technique is safer than the without cuff technique.

**Table-II:** Comparison of Operative and Post-operative Characteristics.

Variable	Cuff Group (Group I)	Without Cuff (Group II)	p-value
Bypass Time (min.) (mean±S.D)	158.56 ± 30.29	135.29 ± 28.08	0.03
X-Clamp* time (min.) (mean±S.D)	101.28 ± 18.78	88.28 ± 14.37	0.04
Duration of Inotrpic support (hours) (mean±S.D)	12.72 ±12.03	17.29 ± 28.88	0.58
Ventilation time (hours) (mean±S.D)	7.94 ± 3.72	10.14 ± 3.82	0.11
ICU** Stay time (hours) (mean±S.D)	48.00 ± 27.30	44.57 ± 22.78	0.70
Total Chest Drainage (ml) (mean±S.D)	488.89 ± 168.27	1158.2 ± 451.25	<0.0001
Hospital Stay time (days) (mean±S.D)	12.78 ± 3.87	10.88 ± 3.82	0.19
Re-opening (%)	0 (0.00)	1 (7.2)	0.25
In-hospital mortality (%)	0 (0.0)	1 (7.1)	0.44

* X-clamp= Cross clamp,

** ICU= Intensive Care Unit.

## DISCUSSION

The Bentall procedure is a surgical repair of an ascending aortic or aortic root aneurysm in combination with aortic valve disease 1^st^ defined in 1968.^1^ Less commonly, it is used to repair aortic dissection affecting the aortic root and valve. During the procedure, a composite aortic valve graft is used to replace the proximal ascending aorta and aortic valve. The original Bentall technique was associated with the risk of coronary separation, false aneurysm formation, and re-operation.^3^,^11^

Kouchoukos and associates in 1981^2^ made some modifications in original Bentall procedure and described button Bentall procedure. That was designed to prevent pseudo-aneurysm formation at the site of the aortic or coronary suture lines. This technique was more difficult and time-consuming than the original Bentall procedure but was associated with better survivalrates.^11^

Better advancement in surgical techniques, prosthetic materials, and tissue preservation methods have now provided the surgeon with a variety of surgical options to treat aortic root disease.^3^,^12^-^14^ Despite all these advancements, bleeding is still one of the most important complications after aortic root replacement.

Recently, many modifications of the original technique have been proposed to prevent bleeding. Like tandem-suture-lines technique, with interrupted mattress sutures to anchor the prosthesis to the aorto-ventricular junction and a running suture between the prosthesis and the remnants of the supra-annular aortic wall (Copeland technique).^15^

Khanna et al.^16^ described that a purse-string suture reinforced with Teflon pledgets through the aortic wall above the proximal suture line can prevent bleeding. Mohite and associates.^17^ recently reported their experience with the use of interrupted pledget edeverting mattress sutures at the proximal anastomosis, passed through the sewing cuff of the valved conduit and then through an autologous pericardial strip. Chen and colleagues^18^ used a prosthesis that was modified with a mini skirt of Dacron attached to the sewing cuff, which was sutured to the remaining proximal aortic tissue—thereby forming a peri-anastomotic space that is then filled with fibrin glue. Recently Alessandro et al. has described the use of fibrin sealant and imbricated U stitches for the proximal anastomosis and showed significantly low risk of bleeding and better post-operative outcomes using this technique.^19^

This study presents excellent short-term outcomes of patients who underwent modified Button Bentall procedure with cuff formation. Use of native aortic cuff above the coronary ostia helps to control bleeding from the aortic anastomosis. This modification will also decrease tension on coronary ostia anastomotic line. Also fixation of cuff of aorta over the conduit increases pressure inside the wrap, thereby decreasing the possibility of blood accumulation. This technique can prove beneficial in controlling bleeding from the coronary ostia anastomotic site and from the annulus suture line as well.

Modified Bentall technique with cuff is little time consuming than the original Modified Bentall procedure. However it brings significant reduction in post-operative bleeding so the risk of re-opening is reduced resulting in better outcomes after surgery.
